# Estimation of the postoperative fatality window in colorectal cancer surgery

**DOI:** 10.1093/bjsopen/zrae153

**Published:** 2025-01-24

**Authors:** Martin Rutegård, Peter Matthiessen, Jörgen Rutegård, Markku M Haapamäki, Johan Svensson

**Affiliations:** Department of Diagnostics and Intervention, Surgery, Umeå University, Umeå, Sweden; Wallenberg Centre for Molecular Medicine, Umeå University, Umeå, Sweden; Department of Surgery, Faculty of Medicine and Health, Örebro University, Örebro, Sweden; Department of Diagnostics and Intervention, Surgery, Umeå University, Umeå, Sweden; Department of Diagnostics and Intervention, Surgery, Umeå University, Umeå, Sweden; Department of Diagnostics and Intervention, Surgery, Umeå University, Umeå, Sweden; Department of Statistics, Umeå School of Business, Economics and Statistics, Umeå University, Umeå, Sweden

## Abstract

**Background:**

Postoperative death measured 30 days after surgery is a conventional quality metric, whereas intervals up to 90 days are increasingly used, although data-driven time windows have scarcely been investigated.

**Methods:**

The Swedish Colorectal Cancer Registry was used to identify all patients subjected resection for colorectal cancer between 2007 and 2020. All patients were followed up until 180 days after surgery. A join-point statistical hazard model was used to model a declining hazard to a transition point, followed by a stable death rate. This method was subsequently applied to describe postoperative deaths for the entire cohort and subgroups according to tumour location (colon and rectum).

**Results:**

Some 56 096 patients electively operated on for colorectal cancer during the study interval were included, with a 30-day and 90-day fatality of 805 (1.43%) and 1458 (2.60%) patients respectively. The derived postoperative fatality window, after which the death rate transitioned to a stable rate, was 23.8 (95% c.i. 21.5 to 28.2) days after surgery. There was no significant difference in the time window between rectal cancer (22.9 days; 95% c.i. 15.1 to 28.4) and colon cancer (27.3 days; 95% c.i. 21.4 to 31.8) patients (*P* = 0.455). However, postoperative fatality time windows were extended in patients aged at least 80 years and with American Society of Anesthesiologists’ grade III or IV.

**Conclusion:**

The traditional postoperative time window of 30 days was confirmed to be an appropriate metric in elective colorectal cancer surgery when evaluated with a hazards-based statistical framework. Importantly, this time window is influenced by older age and advanced co-morbidity, which could prompt increased vigilance for these patient groups.

## Teaser

A man can die but once, but the surgical community have used different metrics for postoperative fatalities. In this registry-based study, we refined a statistical framework using declining hazards. Applying this to elective colorectal cancer surgery, a data-driven postoperative fatality window at 24 days was established, after which the death rate transitioned to a stable rate.

## Introduction

The conventional metrics for postoperative deaths are 30-day deaths or in-hospital deaths, while an extended interval up to 90 days after surgery has gained traction in recent years^1,2^.

The definition of postoperative death and when it is deemed to occur is important, as benchmarking and quality control depend on professional acceptance, as well as on a measure that reliably and accurately captures the essence of the event^3^.

Although true postoperative death would include only death directly induced by the surgery and its consequences, in practice, defined intervals are in use in which all death is considered due to the operation. Lately, some studies used the 90-day fatality window to adequately capture true surgical death, that is related to operative complications, while not also encompassing deaths from other causes, such as cancer recurrence^4–6^.

Another approach to defining a postoperative time window might be derived from hazard rates, describing postoperative fatality intervals, which may vary between surgical procedures and populations. For instance, one such attempt on hepatectomies showed that the hazard of death levelled out at 80 days after surgery, demonstrating that the traditional 30-day metric underestimates the postoperative death rate in this particular context^7^.

There is a need to further evaluate statistical approaches in defining the postoperative fatality interval across different patient populations.

Therefore, a join-point statistical hazard model was applied to clearly define the postoperative fatality transition based on declining hazards and its use was explored on patients who underwent colorectal cancer resection.

## Methods

### Study design

This is a nationwide retrospective cohort study, approved by the regional Ethical Review Board at Umeå University (dnr 2021-04366). Patients treated with abdominal resection for colorectal cancer between 1 January 2007 and 31 December 2020 were identified using the Swedish Colorectal Cancer Registry, and followed up until 30 September 2021. This registry is continuously checked against the National Cancer Registry for completion, and reporting includes patient demography, surgical details, postoperative course and final pathological assessment, as well as long-term outcomes such as recurrence and survival. The registry defines rectal cancer as an adenocarcinoma of the large bowel within 15 cm of the anal verge, as measured by rigid sigmoidoscopy. The Colorectal Cancer Registry has been validated with an average completeness of 99% and overall agreement between registry and re-abstracted variables at 90% for the interval of 2008–2015^8^. The following variables were retrieved from the registry: age, sex, American Society of Anesthesiologists (ASA) fitness grade, year of surgery, histopathological tumour stage and tumour location, as well as the outcome of interest, occurrence and day of postoperative death.

### Outcome of interest

The primary outcome of interest was to define the early postoperative fatality window after colorectal surgery.

### Statistical analyses

A join-point hazard model (*Supplementary materials*, *Fig. S1*) was formulated, assuming that the hazard rate after the operation, denoted the ‘acute phase death rate’, decreases linearly until a time point, the ‘phase shift time’, where the death rate reaches a plateau and remains constant, coined ‘background death rate’, for 180 days after surgery. This model was evaluated in two ways: first, the empirical hazard, representing the observed risk of death, was plotted beside the model hazard, which reflects the predicted risk based on the statistical model (this comparison demonstrates the differences between actual and predicted data); second, the cumulative empirical hazards with pointwise confidence intervals were plotted along the cumulative model hazards (*Supplementary materials*, *Fig. S2*). The highest mortality rates were taken into account to define the acute phase death rate.

For hazard rates, scaling was set to number of deaths per 1000 patients and day. For descriptive purposes, 30-day as well as 90-day deaths were described, as these measures are commonly used for postoperative deaths; in addition, deaths in the interval 3 to 180 postoperative days were noted, as this was the basis of modelling in the particular data set. For comparative purposes, deaths from 180 to 360 days were also described.

Year of surgery was divided into groups: 2007–2011, 2012–2016 and 2017–2020. This categorization was done to reflect and investigate changes over time, presumably as a consequence of improved patient selection (including lower rates of tumour resection) and perioperative care. Age was also categorized in groups: 0–69 years, 70–79 years and at least 80 years. This partition was made to get a reasonable number of early deaths in each category to be able to estimate the hazard structure. ASA was divided into grades I, II and III–IV, where the latter groups were merged as too few patients with ASA IV were operated on to produce meaningful estimates. When evaluating how ASA affected the hazard structure at different age levels, the patients with ASA I had to be omitted, as there were not enough deaths for robust estimation. Subgroup analyses were performed by dividing the cohort into smaller parts. In a post-hoc sensitivity analysis, patients with metastatic disease (TNM stage IV) were also excluded, and the main analysis was repeated to evaluate whether the postoperative time window was influenced by, in many cases, a different and heterogeneous patient group.

The model parameters were estimated with the maximum likelihood method and inference of *P* values and confidence intervals of the model parameters is based on the likelihood ratio test, which was performed only after the applied model passed an overall significance test (see *Supplementary materials*). All tests were two-sided and a significance level of 5% was used. Constructed confidence intervals (c.i.) have 95% coverage.

Due to the small number of missing values, a complete case analysis throughout was used. The software Matlab v. R2021b (The MathWorks Inc., Natick, MA, USA) was used for model building and parameter estimation. Matlab code is available in the Annex.

## Results

### Patients

The study database numbered 64 433 patients operated on for colorectal cancer in Sweden from 2007 to 2020, inclusive. After the exclusion of emergency surgery, 56 239 electively operated on patients remained. In addition, 143 patients with unclear survival status or survival time due to emigration were removed. The final analysis included 56 096 patients. There were 2% missing data points for the ASA variable and 12 cases with missing ages. Basic clinical and demographic variables are presented in *Table 1*. The patients’ median age was 72 years (interquartile range 64–79), most patients were male (53%) and the majority had an ASA fitness grade of II (55%). Tumour location was colonic in 67% of patients, and 12% of patients had metastatic disease.

**Table 1:** Demographic information regarding patients who underwent elective abdominal resection for colon and rectal cancer in Sweden from 2007 to 2020, inclusive

Demographics	Colon (*n* = 37 568; 67%)	Rectal (*n* = 18 528; 33%)	Total (*n* = 56 096; 100%)
**Sex**			
Male	18464 (49.1)	11262 (60.8)	29726 (53.0)
Female	19104 (50.9)	7266 (39.2)	26370 (47.0)
**ASA**			
I	4697 (12.8)	3421 (18.8)	8118 (14.8)
II	19727 (53.6)	10265 (56.4)	29992 (54.6)
III–IV	12368 (33.6)	4512 (24.8)	16880 (30.7)
**Age (years)**			
0–69	13362 (35.6)	9331 (50.4)	22693 (40.5)
70–79	13973 (37.2)	6430 (34.7)	20403 (36.4)
≥ 80	10225 (27.2)	2763 (14.9)	12988 (23.2)
**Year of surgery**			
2007–2011	12580 (33.5)	6557 (35.4)	19137 (34.1)
2012–2016	13149 (35.0)	6699 (36.2)	19848 (35.4)
2017–2020	11839 (31.5)	5272 (28.5)	17111 (30.5)
**(y)pTNM**			
I	6084 (17.7)	4630 (26.8)	10714 (20.8)
II	12987 (37.9)	4675 (27.1)	17662 (34.3)
III	10899 (31.8)	5628 (32.6)	16527 (32.1)
IV	4313 (12.6)	1866 (10.8)	6179 (12.0)
Complete response	10 (0.0)	449 (2.6)	459 (0.9)
**Fatality status**			
Dead day 0–30	583 (1.6)	222 (1.2)	805 (1.4)
Dead day 0–90	1066 (2.8)	392 (2.1)	1458 (2.6)
Dead day 0–2	45 (0.1)	14 (0.1)	59 (0.1)
Dead day 3–180	1636 (4.4)	580 (3.1)	2216 (4.0)
Censored	35887 (95.5)	17934 (96.8)	53821 (95.9)

Values are *n* (%). ASA, American Society of Anesthesiologists; (y)pTNM, pathological tumour stage with and without neoadjuvant treatment.

Patients operated on for colon cancer were slightly more often female (51%), while fewer women had surgery for rectal cancer (39%). ASA fitness grade levels were generally higher in patients with colon cancer. Older patients were more often selected for surgery in the colon cancer group, where patients aged at least 80 years comprised 27% of patients, in comparison to only 15% of patients with rectal cancer. There were no important differences concerning the year of surgery between groups, while it was more common with an advanced tumour stage in patients with colon cancer.

### Mortality rate

The highest mortality rate was observed between postoperative days 3 and 5 (*Supplementary material*, *Fig. S3*). Consequently, the acute phase death rate was defined at day 3, with a linear decline leading up to the ‘phase shift time.’ For the purpose of estimating model parameters, the first 2 postoperative days were excluded from the analysis.

In the cohort as a whole, 805 (1.43%) and 1458 (2.60%) patients died within 30 and 90 days after surgery respectively. Evaluating the interval 3 to 180 postoperative days, deaths amounted to 2275 (4.06%) patients, while only 59 (0.11%) patients died within 2 days of surgery.

#### Postoperative mortality rate: entire cohort

An overall test was used to assess if there were any differences in the hazard structure between levels in factor variables. All the overall tests for the analyses in this study were statistically significant. The details of the estimates presented below are described in *Table 2*.

**Table 2:** Postoperative death by acute phase, background death rate and phase shift time in relation to various clinically relevant groups

Analysed data and connection to figures	Levels	Acute phase death rate (95% c.i.)	Background death rate (95% c.i.)	Phase shift time (95% c.i.)
Colorectal—overall	Combined	0.92 (0.79–1.01)	0.19 (0.18–0.19)	23.8 (21.5–28.2)
Colorectal	Rectal	0.84 (0.68–1.19)	0.14 (0.13–0.16)	22.9 (15.1–28.4)
	Colon	0.90 (0.80–1.08)	0.20 (0.19–0.22)	27.3 (21.4–31.8)
Colon	2007–2011	1.07 (0.89–1.42)	0.25 (0.23–0.28)	31.4 (21.4–36.7)
	2012–2016	0.79 (0.61–1.07)	0.20 (0.18–0.22)	27.0 (18.9–39.7)
	2017–2020	0.84 (0.57–1.05)	0.16 (0.14–0.17)	22.5 (18.5–41.1)
Colon	Age 0–69	0.28 (0.18–0.56)	0.12 (0.10–0.13)	27.9 (8.3–76.2)
	Age 70–79	0.95 (0.68–1.24)	0.19 (0.18–0.21)	19.8 (14.9–29.0)
	Age 80+	1.62 (1.40–2.10)	0.33 (0.30–0.36)	35.5 (28.7–41.0)
Colon	ASA I	0.39 (0.17–1.31)	0.07 (0.05–0.08)	13.3 (4.2–21.3)
	ASA II	0.57 (0.45–0.72)	0.14 (0.13–0.16)	19.8 (17.0–24.3)
	ASA III–IV	1.55 (1.35–1.76)	0.34 (0.32–0.37)	38.7 (33.8–44.2)
Rectal	2007–2011	1.80 (1.27–2.45)	0.21 (0.18–0.24)	10.1 (7.6–14.4)
	2012–2016	0.90 (1.20–0.62)	0.12 (0.10–0.14)	19.1 (15.6–26.9)
	2017–2020	0.34 (0.18–0.65)	0.10 (0.08–0.12)	23.1 (11.6–46.8)
Rectal	Age 0–69	0.51 (0.23–0.86)	0.07 (0.06–0.09)	9.3 (9.3–17.4)
	Age 70–79	1.38 (0.61–1.93)	0.18 (0.15–0.20)	12.0 (10.4–35.2)
	Age 80+	1.98 (1.47–3.35)	0.27 (0.21–0.35)	43.0 (32.0–60.9)
Rectal	ASA I	0.24 (0.06–0.88)	0.04 (0.01–0.05)	24.4 (8.8–46.2)
	ASA II	0.96 (0.63–1.65)	0.11 (0.10–0.13)	13.2 (9.0–20.7)
	ASA III–IV	1.59 (1.22–2.80)	0.30 (0.26–0.35)	25.8 (13.2–32.6)
ASA II	Age 0–69	0.49 (0.25–0.84)	0.09 (0.07–0.10)	8.0 (6.2–11.7)
	Age 70–79	0.76 (0.55–1.05)	0.13 (0.12–0.15)	14.4 (11.2–19.0)
	Age 80+	1.44 (1.07–1.86)	0.22 (0.19–0.25)	21.8 (18.5–27.4)
ASA III–IV	Age 0–69	0.52 (0.37–0.77)	0.20 (0.15–0.24)	66.9 (31.2–120.7)
	Age 70–79	1.40 (1.11–1.70)	0.30 (0.26–0.33)	27.4 (21.7–32.7)
	Age 80+	2.31 (2.00–2.70)	0.45 (0.40–0.50)	39.8 (34.0–44.9)

The acute phase and background death rates are described by a hazard (deaths per day and 1000 patients), while the phase shift time is described in days; 95% confidence intervals (c.i.) are given. ASA, American Society of Anesthesiologists; calendar years denote year of surgery.

**Fig. 1:**
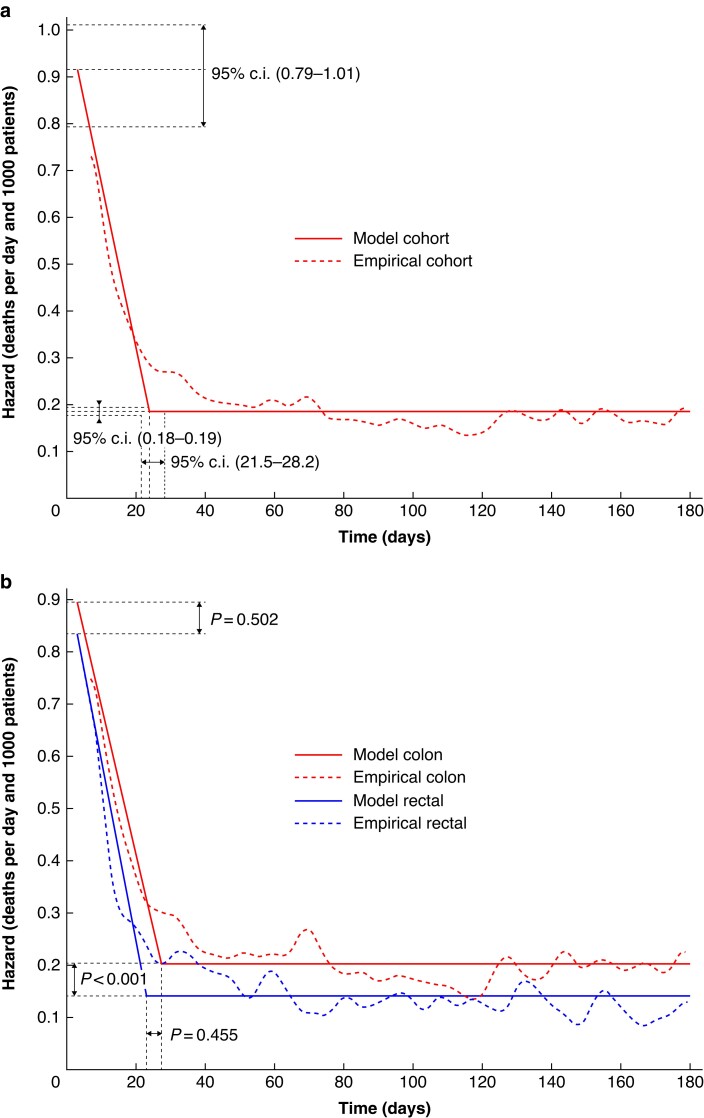
**a Entire study cohort, including elective colon and rectal cancer surgery**. **b Comparison of postoperative deaths between colon and patients with rectal cancer**

The postoperative deaths for the entire cohort, encompassing both elective colon and patients with rectal cancer, are visualized in *Fig. 1*. This figure describes an acute phase where fatality is high but declining rapidly, after which postoperative death transitions into a more stable rate. The empirical curve is not perfectly aligned with the modelled curve, as the former had a smoother transition to the background death rate, thus demonstrating that the phase shift time was not as clearly defined in time as the model suggests. The highest acute phase death rate was 0.917 (95% c.i. 0.794 to 1.012), the phase shift time was 23.8 (95% c.i. 21.5 to 28.2) days and the background death rate was 0.185 (95% c.i. 0.176 to 0.194).

When comparing colon and rectal cancer deaths in *Fig. 1*, there were no significant differences in the highest acute phase death rates (*P* = 0.502), with rates for colon cancer at 0.90 (95% c.i. 0.80 to 1.08) and rectal cancer at 0.84 (95% c.i. 0.68 to 1.19). There were also no differences for the estimated change in the phase shift time (*P* = 0.455), with the estimated day for colon cancer at 27.3 (95% c.i. 21.4 to 31.8) and for rectal cancer at 22.9 (95% c.i. 15.1 to 28.4). The background death rate was significantly higher for patients with colon cancer (*P* < 0.001), with rates for colon cancer at 0.20 (95% c.i. 0.19 to 0.22) and rectal cancer at 0.14 (95% c.i. 0.13 to 0.16). When excluding stage IV patients in a sensitivity analysis, the phase shift time amounted to 27.5 days (95% c.i. 24.9 to 30.0) after surgery (*Supplementary material*, *Fig. S4*). Death 180–360 days after surgery was approximately constant in time (*Supplementary material*, *Fig. S5*).

#### Postoperative mortality rate: colon cancer

**Fig. 2:**
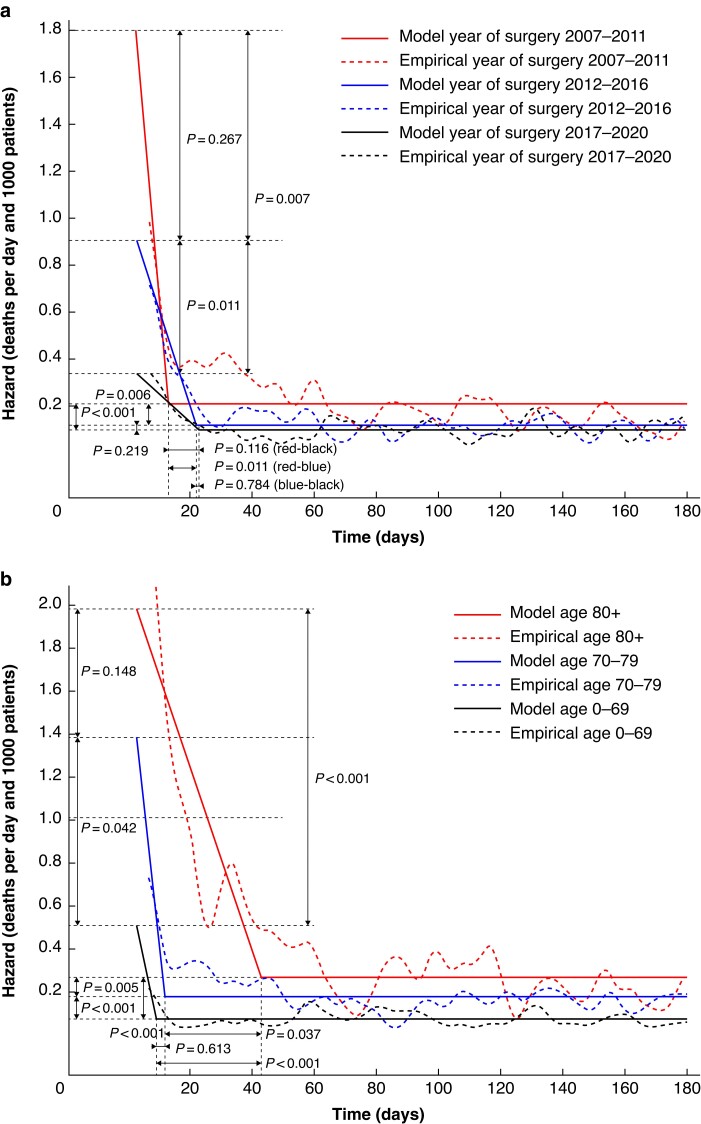

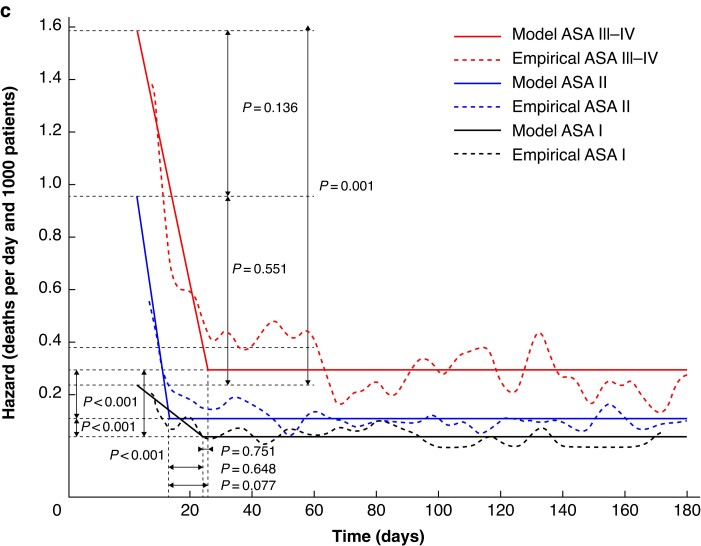
Postoperative deaths in patients with colon cancer by: a year of surgery, b age groups, c American Society of Anesthesiologists’ (ASA) fitness grade

The postoperative mortality rate in patients with colon cancer by year of surgery is visualized in *Fig. 2*. Here, no changes in the acute phase death rate nor the phase shift time could be discerned between time intervals. However, operations performed in the later intervals had a lower background death rate.

In *Fig. 2*, postoperative death as a function of age is described. For patients with colon cancer, there were substantial differences in the acute phase and the background death rates for all age groups, as older patients experienced higher death rates. The oldest age group (80 years and older) had a significantly larger phase shift time than the group aged 70–79 years.

In *Fig. 2*, postoperative death as a function of ASA fitness grade is presented. For patients with colon cancer, there were significant differences in the acute phase death rate between the most co-morbid group of ASA III–IV patients and the other groups. There were also significant differences in the background death rate between all ASA categories, where a higher ASA fitness grade was associated with higher hazard rates. The phase shift time for patients with ASA III–IV was significantly higher compared with patients with lower grades, where ASA I patients had 13.3 days (95% c.i. 4.2 to 21.3), ASA II 19.8 days (95% c.i. 17.0 to 24.3) and ASA III–IV had 38.7 days (95% c.i. 33.8 to 44.2).

#### Postoperative mortality rate: rectal cancer

**Fig. 3:**
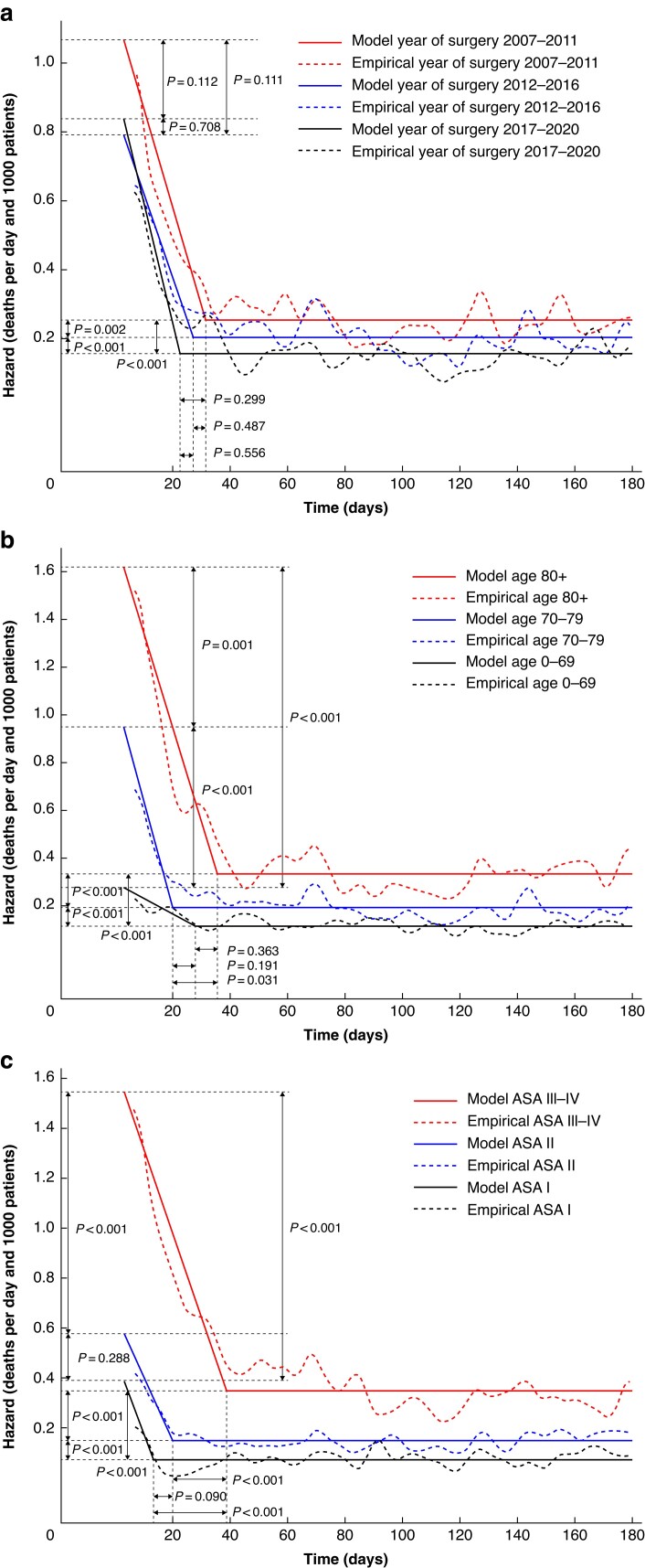
Postoperative deaths in patients with rectal cancer by: a year of surgery, b age groups, c American Society of Anesthesiologists’ (ASA) fitness grade

The postoperative mortality rate in patients with rectal cancer by year of surgery is visualized in *Fig. 3*. Here, we can detect a consistent decrease in the acute phase death rate between 2007–2011 and 2017–2020 (*P* = 0.007) as well as between 2012–2016 and 2017–2020 (*P* = 0.011). There is also a detectable change in phase shift time between 2007–2011 and 2012–2016 (*P* = 0.011). The background death rate was lower for later intervals with discernible changes between 2007–2011 and 2012–2016 (*P* = 0.006) as well as between 2007–2011 and 2017–2020 (*P* < 0.001). Notably, there is a major lack of fit for the 2007–2011 line as the transition to the background death rate is not as sharp as the parametric model dictates. In *Fig. 3*, postoperative deaths as a function of age is described. Higher age was associated with a higher acute phase death rate maximum with significant changes between age groups less than 70 years and 80 years or older (*P* < 0.001) and between less than 70 years and 70–79 years (*P* = 0.042). The highest age group 80 years or older was associated with a larger phase shift time at 43 (95% c.i. 32.0 to 60.9) compared with ages less than 70 years (*P* < 0.001) and ages 70–79 years (*P* = 0.037). Lower age was associated with a lower background death rate. The oldest age group displayed a smooth, not clearly defined, transition to the background death rate.

In *Fig. 3*, the postoperative mortality rate as a function of ASA fitness grade is also presented. For patients with rectal cancer, there was a discernible difference in the acute phase death rate between patients with ASA I and ASA III–IV, as the latter group had a significantly higher acute phase death rate. Moreover, the lower the ASA fitness grade, the lower the background death rate became. There were no significant differences in the phase shift time between ASA levels.

### Impact of ASA in different age groups

**Fig. 4:**
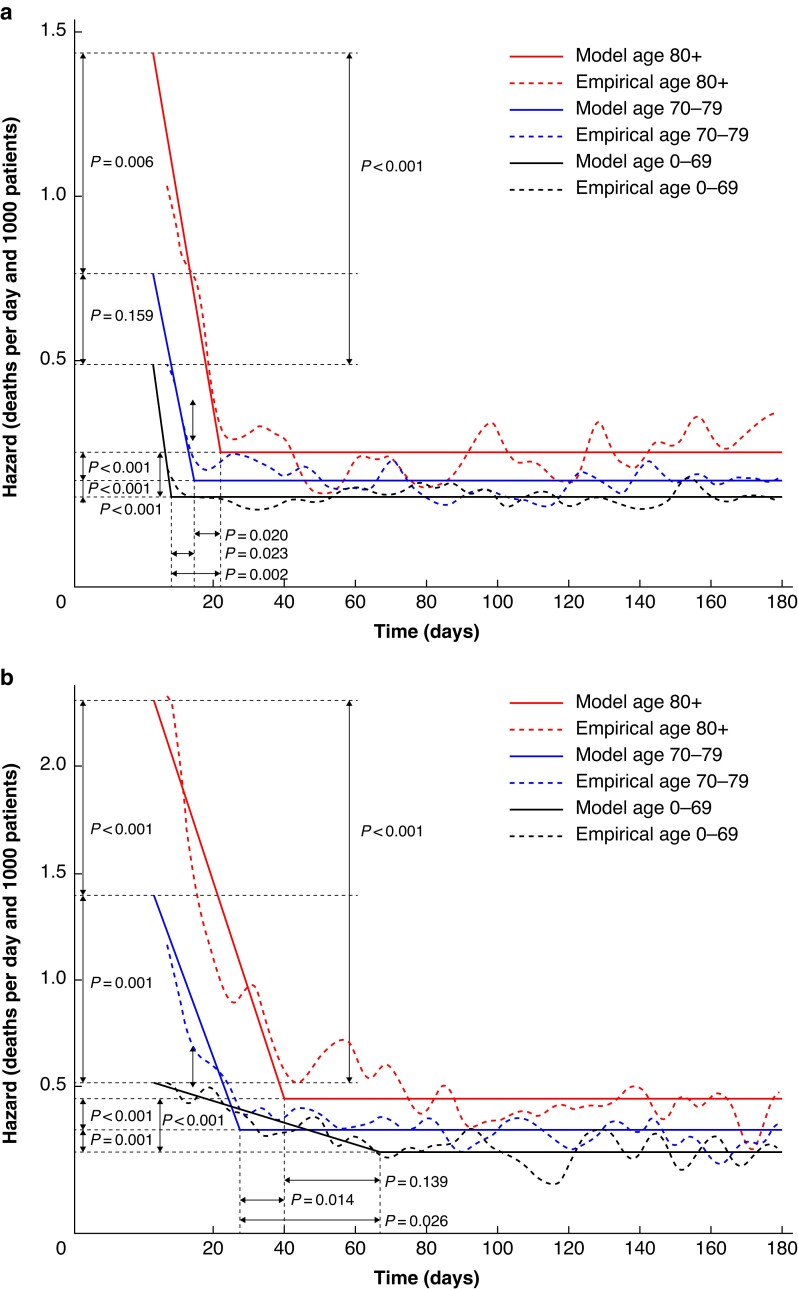
Postoperative deaths in patients with colorectal cancer by age among: a American Society of Anesthesiologists’ (ASA) fitness grade II patients, b ASA grade III−IV patients

Since ASA and age are related variables, subgroup analyses were conducted for different age groups within ASA II as well as within ASA III–IV (*Fig. 4*). Of note, there were discernible differences across age groups within each ASA level, suggesting an independent impact of age on estimated deaths.

## Discussion

In a nationwide cohort of elective colorectal cancer resection surgery, a join-point statistical hazard model was used to define the postoperative deaths transition to a point, whereafter a stable death rate ensued. This postoperative fatality window, the time with an increased hazard, was found to last 24 days after surgery. However, this was dependent on patient-related variables such as age and ASA fitness grade, as well as structural factors such as year of surgery. For instance, the sensitivity analysis demonstrated that exclusion of patients with metastatic disease slightly extended the postoperative time window to 28 days. Higher age was shown to be independently associated with death phase rates irrespective of ASA fitness grade, exemplifying control for confounding with stratification within the statistical model.

This study has several weaknesses. To facilitate modelling and interpretability, a model with a hazard decline followed by a stable baseline hazard was imposed. Not only was it necessary to exclude the first 2 postoperative days from this model to improve reliability, but there were also obvious discrepancies when comparing the models to the empirical hazards. While this inevitably leads to uncertainties for the derived estimates, this model also does not allow for other plausible shapes for the postoperative hazards. Although the derived model generally worked appropriately, there were instances of major lack of fit. Nevertheless, this modelling allowed for an acute phase of high but declining mortality rate, corresponding to an immediate postoperative fatality interval, while the decline to the background death rate marked the end of the former interval and allowed for a stable mortality rate, expected after surgical recovery. Another strength of the present study is the nationwide and therefore, population-based design, alleviating selection bias and improving external validity.

Metrics other than the traditional 30-day measure have gained popularity, with most researchers using 90-day postoperative deaths as a new reference, although just as arbitrarily chosen as the former^2,9^. In a single-centre American study, cause of death was established using charts of colorectal patients, noting that up to about 90 days after surgery, the vast majority of deaths were due to postoperative complications, while this constituted a rare cause thereafter; moreover, predictors for death were similar in regression models with 30- as well as 90-day deaths as the outcome, whereas modelling with later occurring deaths revealed a substantially different predictor set^6^. In a large registry-based study using nationwide English data, it was shown that patients below 65 years of age exhibited a high rate of cardiac deaths also in the 90-day time interval^10^. In a more formal way, though evaluating oesophageal cancer postoperative deaths, Dutch researchers investigated the diagnostic properties of a 30-day and 90-day metric. Postoperative timing and cause of death was established in a standardized manner, where accuracy for a postoperative complication leading to death was measured; it was shown that the 90-day metric performed substantially better, with a large gain in identifying true postoperative deaths^5^. In a similar study of hepatobiliary and pancreatic surgery, American researchers derived time windows ranging from 99 to 118 days, which optimally reflected surgically related death^4^. The present study is different from these studies, as it used hazard rate estimations exclusively to derive the postoperative time window of interest. This window was estimated to be 24 days, which is an even shorter time frame than the traditional 30-day metric, and the present study thus lends no real credence to extending this interval to 90 days after colorectal cancer surgery. As the nature and cause of deaths were not investigated in the present study, comparisons to some of the chart-based literature is difficult; it might simultaneously be true that the mortality rate itself declines quite quickly after surgery, and that complications related to surgery induce death later on (impacting the background death rate, using the terminology of the present study). In contrast, a hazards-based approach for threshold estimation has been developed^11^ and tested on a partial hepatectomy cohort, where it was shown that the hazard rate of death levelled out at 80 days, also well beyond the 30-day mark^7^. This method is similar but not equal to the one used in the present study, and a probable reason for this discrepancy is the different patient populations and procedures involved.

The methodology for the determination of a postoperative time window can be used to establish specific benchmarks for a wide range of populations and procedures. It is expected that these time windows differ across operations, as a function of the surgical trauma and the patient population. Establishing more procedure-specific postoperative time windows is important to surgeon and patient alike, informing clinical decision-making as well as clinical research; the traditional 30-day metric is partially inadequate in this respect, as for some procedures this measure might capture only a small share of the deaths which should be attributed to the operation, while the reverse is true for many others. In addition, nowadays even major surgical procedures, for example abdominal resection of rectal cancer, typically convey such a small 30-day fatality risk that predicting death is increasingly difficult^12^. In the present study, several examples with differing mortality rates according to time interval, age and ASA fitness grade are presented. These might be important in clinical scenarios, as for example higher age in rectal cancer surgery imparts a decidedly longer postoperative fatality time window, potentially prompting higher vigilance even after discharge in this age group compared with younger patients. Evaluation of intervals can also be used to monitor fatality changes over the years, not easily captured by a single metric such as 30-day or 90-day deaths.

## Supplementary Material

zrae153_Supplementary_Data

## Data Availability

Data can be shared subject to approval from the steering committee of the Swedish Colorectal Cancer Registry.
