# Supplementary material for: Estimation of the postoperative fatality window in colorectal cancer surgery
Source: BJS Open. 2025 Jan 24;9(1):zrae153. doi: 10.1093/bjsopen/zrae153 (PMC11758370; doi:10.1093/bjsopen/zrae153)
Supplement: zrae153_Supplementary_Data [file zrae153_supplementary_data.zip › Supplementary_Material.docx]

# Supplementary Material for article “Estimation of the postoperative mortality window in colorectal cancer surgery”

## Authors

Martin Rutegård, MD, PhD (1,2), Peter Matthiessen, MD, PhD (3), Jörgen Rutegård, MD, PhD, FRCS (1), Markku M Haapamäki, MD, PhD (1), Johan Svensson, PhD (1, 4).

## Affiliations

1) Department of Diagnostics and Intervention, Surgery, Umeå University, Umeå, Sweden; 2) Wallenberg Centre for Molecular Medicine, Umeå University, Umeå, Sweden; 3) Department of Surgery, Faculty of Medicine and Health, Örebro University, Örebro, Sweden; 4) Department of Statistics, Umeå School of Business, Economics and Statistics, Umeå University, Umeå, Sweden.

## Corresponding author

Martin Rutegård ([martin.rutegard@umu.se](mailto:martin.rutegard@umu.se))

Department of Diagnostics and Intervention, Surgery, Umeå University

SE-901 85 Umeå, Sweden

ORCID ID 0000-0002-0974-6373

Twitter: @martin_rutegard

##

## Supplementary Materials - Index

| **Supplementary Methods** |  |
| --- | --- |
| Postoperative hazard model formulation | *pag. 3* |
| Hazard structure | *pag. 3–5* |
| Point estimation | *pag. 5–6* |
| P values and confidence intervals | *pag. 6–7* |
| Overall test | *pag. 7–8* |
| Evaluation of model fit | *pag. 8* |
| Some notes on model development | *pag. 10–11* |
| **Supplementary Figures** |  |
| Figure S1 | *pag. 4* |
| Figure S2 | *pag. 9* |
| Figure S3 | *pag. 11* |
| Figure S4 | *pag. 12* |
| Figure S5 | *Pag 13* |
| **References** | *pag. 14* |

## Postoperative hazard model formulation

Joinpoints or changepoints are typically estimated using methods such as segmented regression(1-3) or maximum likelihood estimation (MLE)(4, 5). In this study, we estimate a joinpoint that describes the phase transition to baseline mortality following colorectal cancer resection. This estimation is based on an MLE approach, which utilizes a parametrization of the hazard function and the standard survival likelihood for right-censored data. In the appendix, we initially outline our method for parameterizing the hazard function using three pertinent features. These features are further parameterized using patent characteristics. We then formulate a likelihood and describe the parameter inference based on the MLE and likelihood ratio test. Additionally, we explain our approach to evaluating the model.

### Hazard structure

We suggest a model to model the hazard in a fixed time window after surgery. The model specifies a hazard structure with three features. The hazard rate after operation, denoted *a*, is labelled “acute phase death rate”, and decreases linearly until a time point, denoted $t_{p}$, named “phase shift time”, where the death rate remains constant, denoted *b*, which is called “background death rate” (Figure S1). From the model we can estimate how patient characteristics influence the phase transition from the operation related death rate to the background death rate.


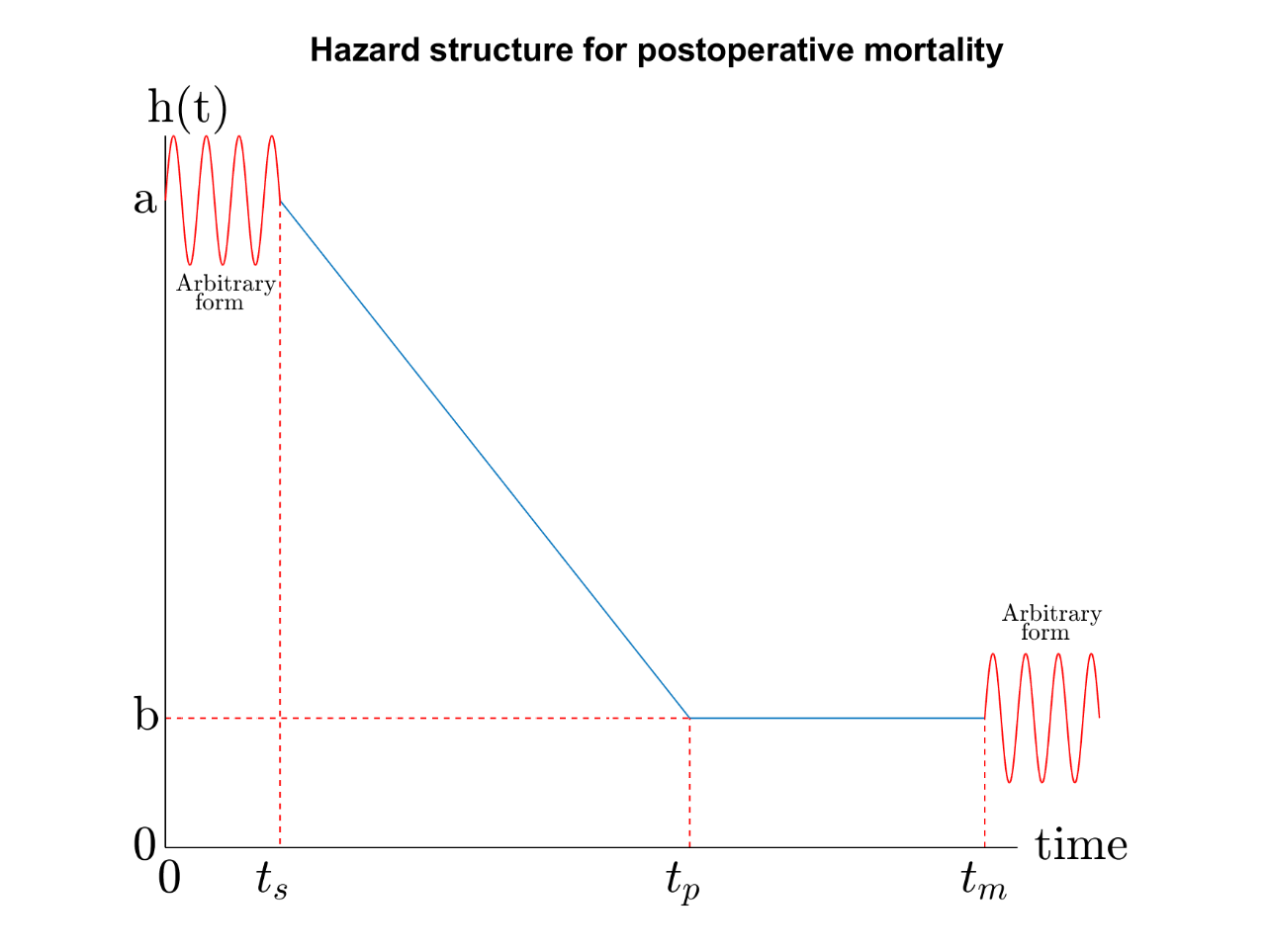


**Figure S1:** Hazard structure for postoperative mortality where *a* represents the acute phase death rate, *b* represents the background death rate and $t_{p}$ represents the phase shift time. The model starts at time point $t_{s}$ and stops at time point $t_{m}$.

We start to model the hazard at time point $t_{s}$(start) after operation and stop at $t_{m}$ (max). The time points $t_{s}$ and $t_{m}$ are set by the user to capture desired properties of the hazard while maintaining model fit.

The hazard form described in Figure S1 is parameterized as

$$h\left( t \right)=\left( a-\frac{a-b}{t_{p}-t_{s}} (t-t_{s}) \right) I\left\{ t_{s}<t\leq t_{p} \right\}+b I\left\{ t_{p}<t\leq t_{m} \right\}$$

with the following cumulative hazard

$$H\left( t \right)=\left( a(t-t_{s})-\frac{a-b}{t_{p}-t_{s}} \frac{\left( t-t_{s} \right)^{2}}{2} \right) I\left\{ t_{s}<t\leq t_{p} \right\}+\left( \frac{a+b}{2}(t_{p}-t_{s})+b(t-t_{p}) \right) I\left\{ t_{p}<t{\leq t}_{m} \right\}$$

where $I\{\cdot\}$ is an indicator function given value 1 when expression $\{\cdot\}$ is true, else 0. We define hazard parameters $a$ and $b$ to be positive and $t_{p}>t_{s}$.

Next, we incorporate patient characteristic data in the model by the parametrization,

$a=e^{a_{0}+a_{1}x_{1}+a_{2}x_{2}+\ldots+a_{p}x_{p}}$,

$b=e^{b_{0}+b_{1}x_{1}+b_{2}x_{2}+\ldots+b_{p}x_{p}}$,

$t_{p}-t_{s}=\tau=e^{\tau_{0}+\tau_{1}x_{1}+\tau_{2}x_{2}+\ldots+\tau_{p}x_{p}}$,

where $x_{i}, i=1,\ldots,p$ are patient characteristics and ${a_{i},b_{i},\tau}_{i} i=1,\ldots,p$related parameters. The parametrization keeps the hazard parameters $a$, $b$ and $\tau$ positive regardless of values of patient characteristics.

### Point estimation

Patients deceased or censored before the starting time point $t_{s}$, do not provide information to the hazard structure and are removed. For the remaining observations we have the time vector $\boldsymbol{t}=(t_{1},\ldots,t_{n})$connected to a vector of indicators $\boldsymbol{d=}{(d}_{1}\boldsymbol{,\ldots,}d_{n}\boldsymbol{)}$for death $(d_{i}=1)$ and censoring $(d_{i}=0)$, and the likelihood for the parameters $\boldsymbol{\theta}= (a_{0},\ldots,a_{p},b_{0},\ldots,b_{p}, \tau_{0},\ldots,\tau_{p})$ is

$$L\left( \boldsymbol{\theta}\mid\boldsymbol{t},\boldsymbol{d} \right)=L(\boldsymbol{\theta})= \prod_{\{i:d_{i}=1\}} f\left( t_{i};\boldsymbol{\theta} \right) \prod_{\{i:d_{i}=0\}} S\left( t_{i};\boldsymbol{\theta} \right)=\prod_{\{i:d_{i}=1\}} h(t_{i};\boldsymbol{\theta})\prod_{i=1}^{n} e^{-H(t_{i};\boldsymbol{\theta})}$$

with density function $f\left( t \right)=h\left( t \right)e^{-H\left( t \right)}$ and survival function$S\left( t \right)=e^{-H\left( t \right)}$. Times larger than $t_{m}$ are defined censored at time point $t_{m}$. Let $l\left( \boldsymbol{\theta} \right)=\log\left( L\left( \boldsymbol{\theta} \right) \right)$ be the log likelihood. Then the maximum likelihood point estimate is $\hat{\theta}=\underset{\theta\in\Theta}{\mathrm{argmax}} l(\boldsymbol{\theta})$ with given data $\boldsymbol{t}$ and $\boldsymbol{d}$ in the unrestricted parameter domain $\Theta$.

**Comment 1**: For interpretation of categorical patient variables, we suggest transforming patient characteristics, parameters $a_{i} , b_{i}$ and $\tau_{i}$ to hazard parameters *a, b* and $t_{p}$ for the actual category. This will facilitate interpretation and is done in Table 2. We suggest categorization of continuous covariates to simplify interpretation.

**Comment 2:** The model has many options to apply extra constraints. For instance, one can assume a constant background death rate that does not change with the patients’ characteristics. This is equivalent to the estimating the parameter constraining the domain $\Theta$ with $b_{i}=0, i=1,..,p$.

### P values and confidence intervals

We use the likelihood ratio test to calculate p values and confidence intervals for single parameters(6, 7). Of particular interest is p values for the parameters $a_{i} , b_{i} , \tau_{i}$ when $i=1,\ldots,p$. Those parameters describe if patient characteristics are related to change of the hazard features. Without loss of generality, we here describe the procedure for the hypothesis $H_{0}:a_{1}=0$ vs $H_{A}:a_{1}\neq0$. Inference for other parameters is identical. Let $\hat{\theta}_{0}=\underset{\theta\in\Theta:a_{1}=0}{\mathrm{argmax}} l(\boldsymbol{\theta})$ be the constrained ML-estimate under $H_{0}$. The likelihood ratio test statistic $X_{LR}^{2}=2\left[ l\left( \hat{\theta} \right)-l\left( \hat{\theta}_{0} \right) \right]$is then $\chi^{2}$ distributed with one degree of freedom under $H_{0}$ and the corresponding p value the area to the right of the $X_{LR}^{2}$ in the $\chi^{2}$ distribution. The connected confidence interval is achieved by profiling $l_{P}\left( \tilde{a}_{1} \right)=\max_{\theta\in\Theta:a_{1}=\tilde{a}_{1}} l(\theta)$ and is given by $CI_{a_{1}}^{95\%}=[a_{1}\mid2 \left( l\left( \hat{\theta} \right)-l_{P}\left( a_{1} \right) \right)\leq\chi_{1,95\%}^{2}]$ where $\chi_{1,95\%}^{2}=3.84$ is the 95% quantile of the $\chi^{2}$ distribution with one degree of freedom.

In Table 2, confidence intervals for the hazard features $e^{a_{0}}, e^{b_{0}}$ and $e^{\tau_{0}}$ for the reference category are presented and derived by formulas above, changing reference category in parametrization. The likelihood-based confidence intervals are invariant to one-to-one variable transformations, as discussed in Neale et al(8).

### Overall test

For each patient characteristic added to the model, three new parameters are estimated – one for each feature of the defined hazard structure. Before proceeding to inference for individual hazard features, we suggest an overall test to answer the question: are the selected patient characteristics related to a change in the hazard structure? We formulate the following statistical hypothesis:

$H_{0}:$ The patient characteristics do not change the hazard structure
⇔ $a_{i}=b_{i}=\tau_{i}=0, i=1,\ldots,p$

$H_{A}:$ The patient characteristics do change the hazard structure
⇔ ${At least one of a}_{i}\neq0 or b_{i}\neq0 or \tau_{i}\neq0 for some i=1,\ldots,p$
We resolve the hypothesis inference by using the likelihood ratio test. We let $\hat{\theta}_{0}=\underset{\theta\in\Theta:\theta=\theta_{0}}{\mathrm{argmax}} l(\boldsymbol{\theta})$ be the constraint ML-estimate under $H_{0}$ where all parameters except $a_{0}, b_{0}$ and $\tau_{0}$ are set to zero and $\hat{\theta}=\underset{\theta\in\Theta}{\mathrm{argmax}} l(\boldsymbol{\theta})$ be the unconstrained maximization.
$X_{LR}^{2}=2\left[ l\left( \hat{\theta} \right)-l\left( \hat{\theta}_{0} \right) \right]$is then $\chi^{2}$ distributed with $3p$ degrees of freedom and corresponding p value the area to the right of the of $X_{LR}^{2}$ in the $\chi^{2}$ distribution.

**Comment 3:** All the overall tests used in the article had significant test results. Most often, the feature background death rate differed between different patient groups. Although the change in background death rate was small between patient groups, this difference was easy to detect because of the high precision of the estimate based on many individuals during a long time period. Change of the acute phase death rate and the phase shift time were harder to detect, since the precision of those estimates was determined by the number of deaths in a smaller time window, closer to operation.

### Evaluation of model fit

For categorical patient characteristics, we visually examine subgroups defined by the categorical characteristics by comparing the model hazard with the empirical hazard. Firstly, we estimate the cumulative hazard with the Nelson Aalen estimator(9) with connected pointwise confidence intervals. Secondly, we used a smoothed version of the empirical hazard based on a gaussian shaped kernel applied on the increments of the Nelson Aalen estimator. The model-based hazard and cumulative hazards were plotted alongside the empirical Nelson Aalen estimates. A common failure of the specified hazard structure is that the transition to the baseline phase death rate is smoother than the model suggests. In Figure S2, we see an example of such a lack of fit for the entire colorectal group.


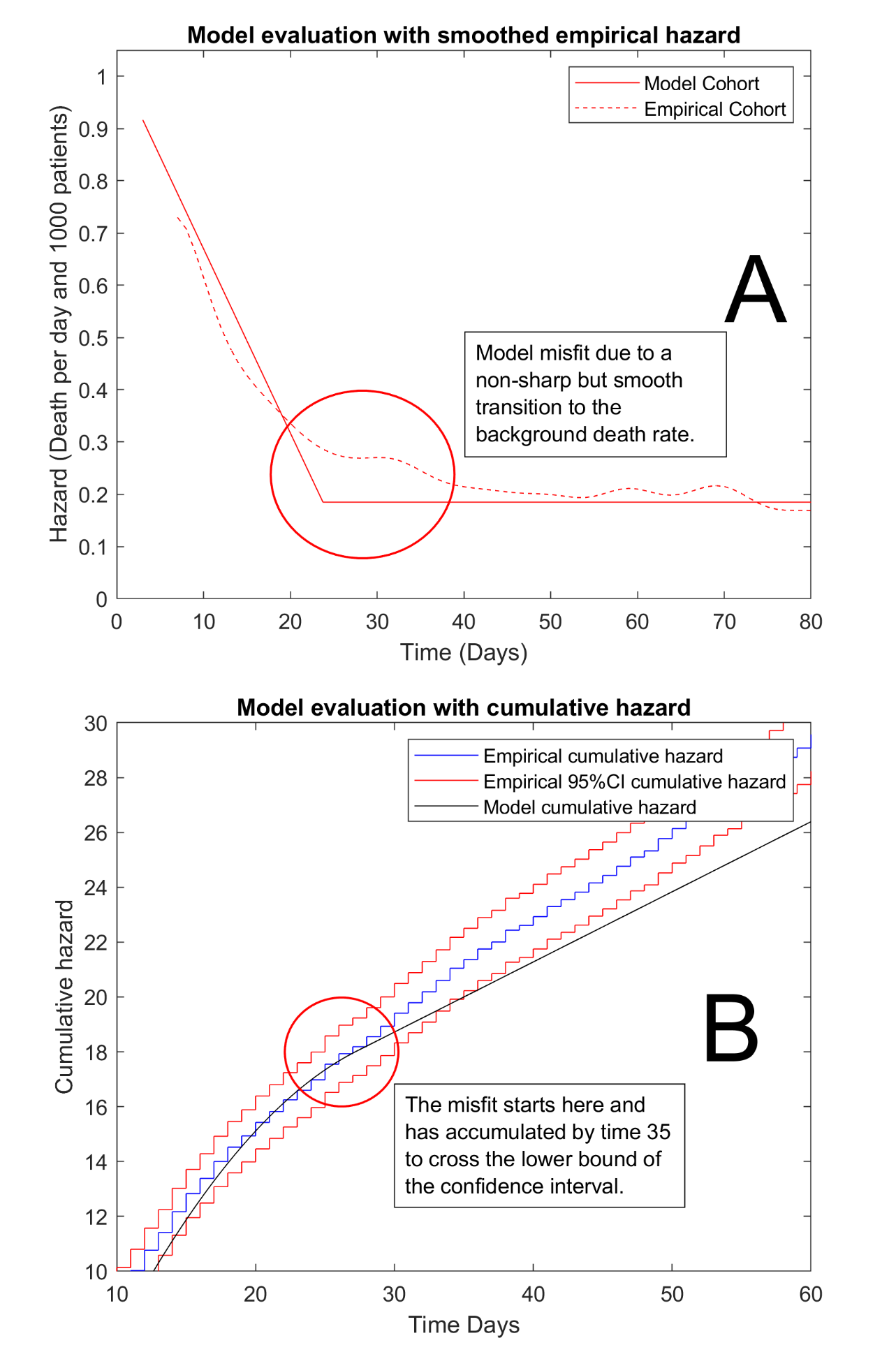


**Figure S2:** Model evaluation with A: a smoothed empirical hazard function plotted alongside the model hazard function. A non-sharp, smooth transition to background death rate exemplifies model misfit. Panel B illustrates the empirical cumulative hazard function with 95% pointwise confidence intervals, used for model evaluation on the same data.

### Some notes on model development

Our aim was to estimate and evaluate how different characteristics affected the phase shift time. While plotting the data, we observed an almost linear decline of death rate after surgery to a time point where the death rate levelled out. A mathematical description of this pattern becomes our first model.

**Main model structure:** In this first analysis attempt, we modelled the baseline phase rate as a slowly decaying function with a slope. However, this slope was usually inseparable from zero, indicating a constant background death rate from the phase shift time to 180 days after surgery. For some scenarios, the baseline death rate slope parameter captured unwanted characteristics in data when there was no clear phase shift time, but rather a slower decay to the background death rate. We finally decided to remove the background death rate slope and let the background death rate be constant between the phase shift time to 180 days after surgery. This gave a better estimate of the phase shift time in cases where model lack of fit was an issue.

**Patient characteristic structure:** When adding patient characteristics to the model, at first we only assumed that the phase shift time depended on the characteristic. The acute phase death rate and the background death rate was considered the same for all patient groups. This approach led to serious model misfit since the acute phase death and the background death rates were heavily affected by the patient characteristics. After consideration, we added the ability for patient characteristics to change both the acute phase death rate and the background death rate.

**Death shortly after surgery:** In early model stages, we assumed that the hazard rate was highest at the time of operation; however, this turned out to be incorrect. The peak mortality is somewhere between days 3 to 5 and is stable in different subsets of the data; we chose to use day 3 as a cut-off to preserve as many deaths in the cohort as possible, while still retaining acceptable stability. There is a decrease in mortality at day 2 and earlier. At the day of surgery (day 0) the patients do not experience 24 hours of risk and the expected number of dead patients should be somewhat lower (Figure S3).


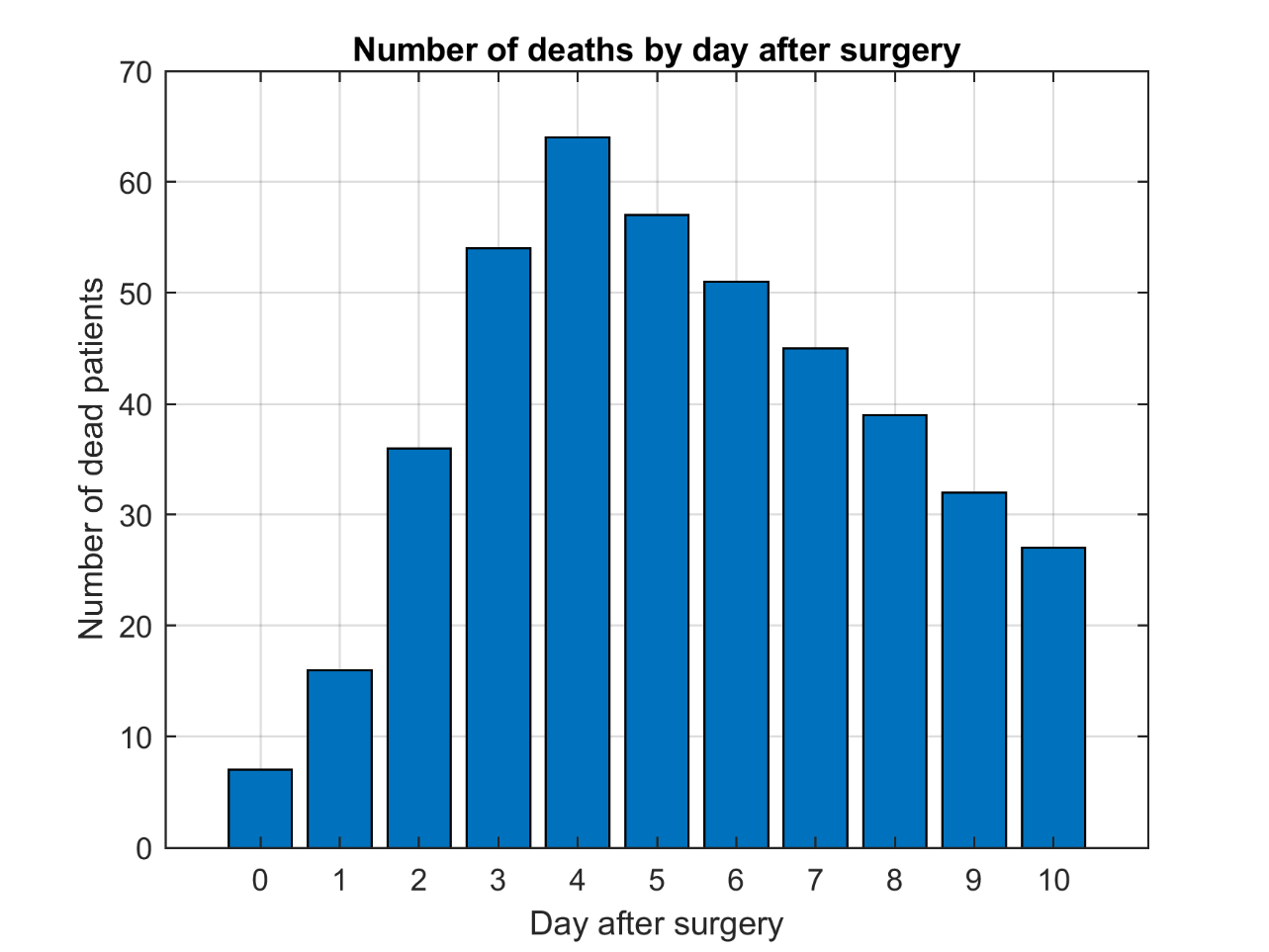


**Figure S3**: Number of deaths by day up to 10 days after surgery for 56 thousand cases of elective colorectal cancer surgery.


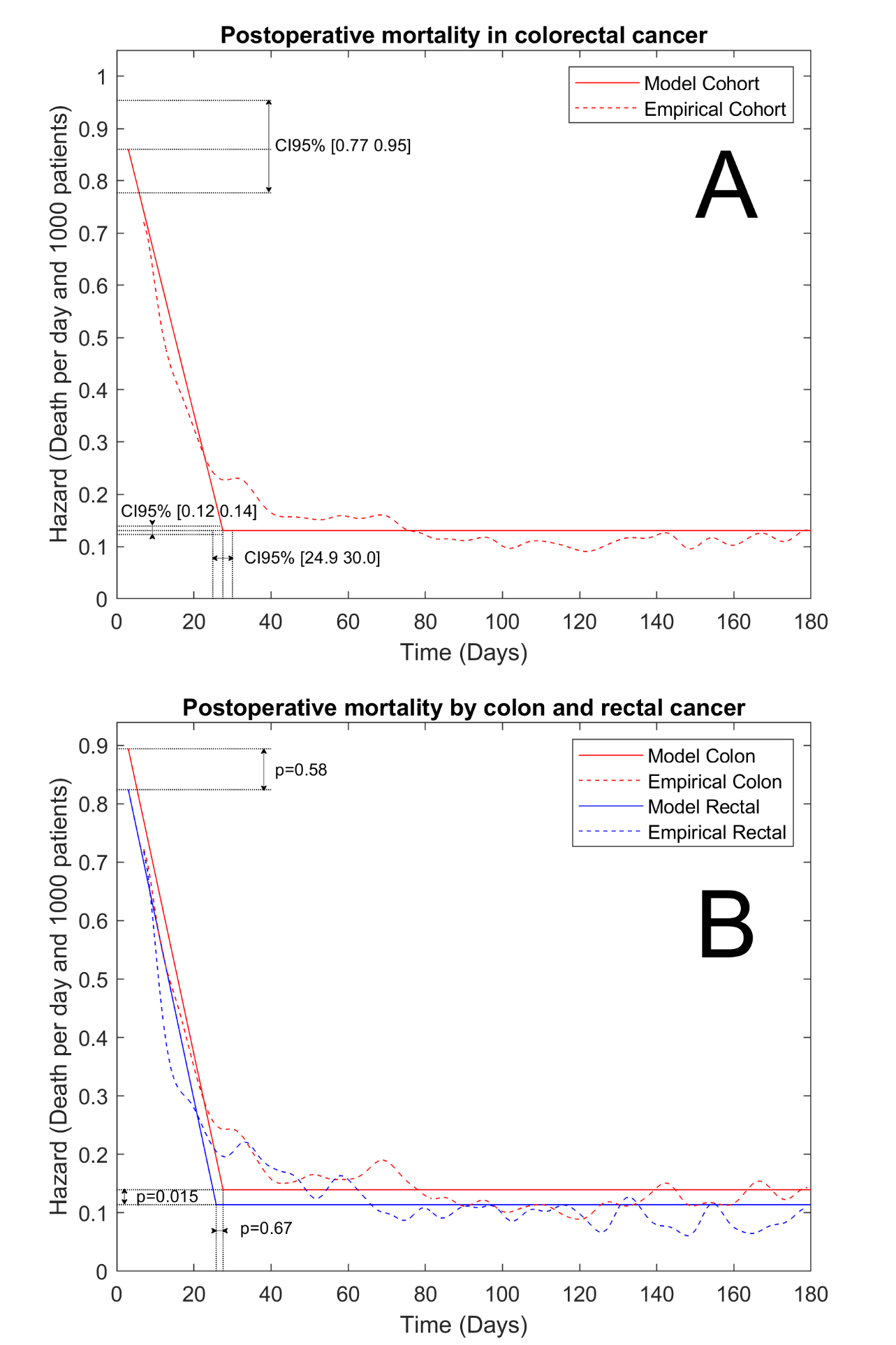


**Figure S4:** Exclusion of stage IV patients. A: Entire study cohort, including elective colon and rectal cancer surgery. B: Comparison of postoperative mortality between colon and rectal cancer patients.


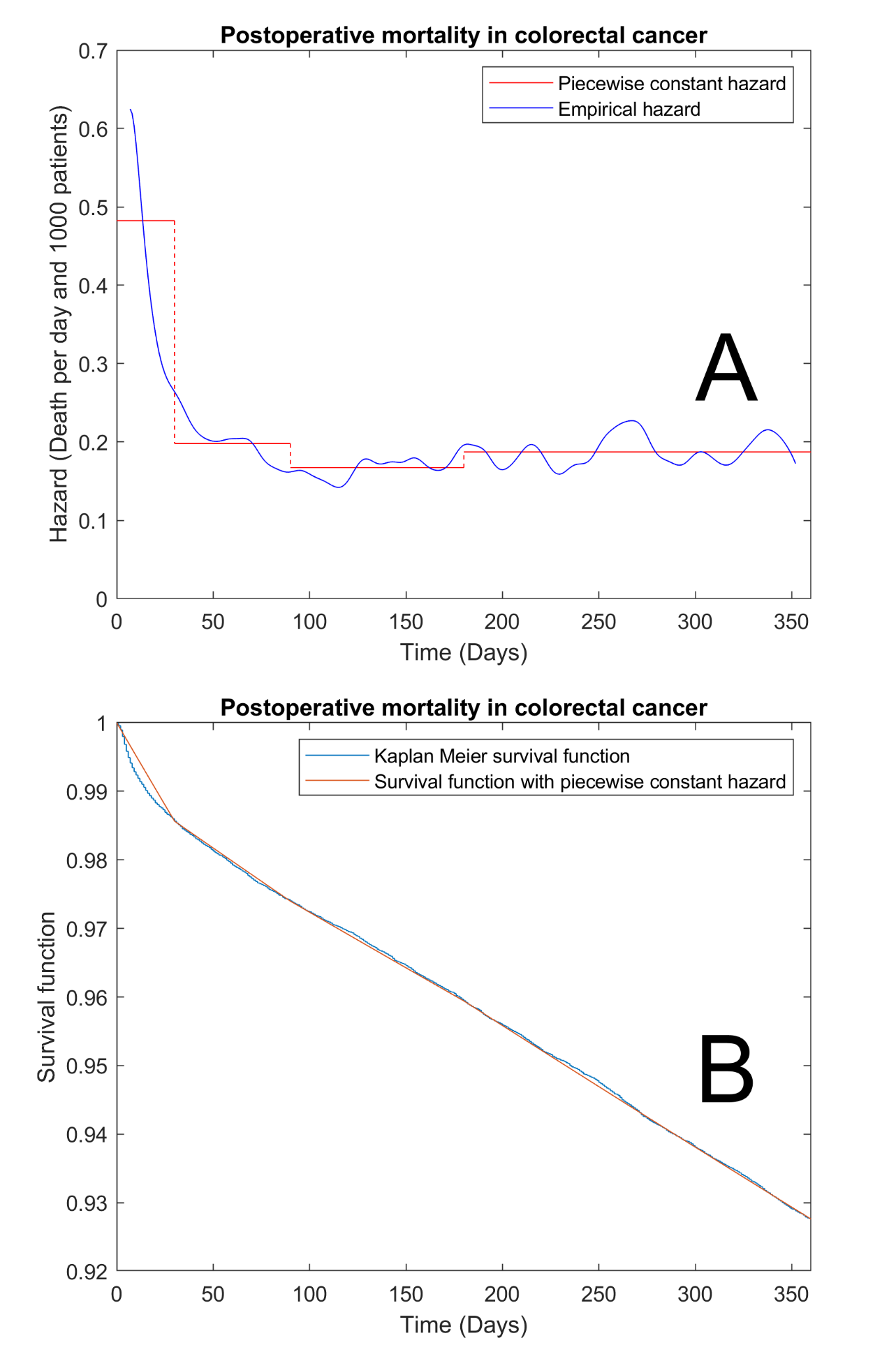


**Figure S5:** Postoperative mortality within 360 days after colorectal cancer resection, approximated with a piecewise constant hazard. Intervals used are 0–30, 31–90, 91–180 and 181–360 days. Illustrated with a hazard plot A and a survival function plot B. Note that the vertical axis starts at 92% in plot B.

## References

1. Dragomirescu I, Llorca J, Gomez-Acebo I, Dierssen-Sotos T. A join point regression analysis of trends in mortality due to osteoporosis in Spain. Sci Rep. 2019;9(1):4264.

2. Rea F, Pagan E, Compagnoni Monzio M, Cantarutti A, Pugni P, Bagnardi V, Corrao G. Joinpoint regression analysis with time-on-study as time-scale. Application to three Italian population-based cohort studies. Epidemiology, Biostatistics, and Public Health. 2017;14(3).

3. Muggeo VM. Estimating regression models with unknown break-points. Stat Med. 2003;22(19):3055-71.

4. Li YX, Qian LF, Zhang W. Estimation in a change-point hazard regression model with long-term survivors. Stat Probabil Lett. 2013;83(7):1683-91.

5. Yu B, Huang L, Tiwari RC, Feuer EJ, Johnson KA. Modelling Population-Based Cancer Survival Trends by using Join Point Models for Grouped Survival Data. Journal of the Royal Statistical Society Series A: Statistics in Society. 2009;172(2):405-25.

6. Fischer SM, Lewis MA. A robust and efficient algorithm to find profile likelihood confidence intervals. Statistics and Computing. 2021;31(4):38.

7. Venzon DJ, Moolgavkar SH. A Method for Computing Profile-Likelihood-Based Confidence-Intervals. J R Stat Soc C-Appl. 1988;37(1):87-94.

8. Neale MC, Miller MB. The use of likelihood-based confidence intervals in genetic models. Behav Genet. 1997;27(2):113-20.

9. Aalen O. Nonparametric Inference for a Family of Counting Processes. The Annals of Statistics. 1978;6(4):701-26.
