# Supplementary material for: Estimation of the postoperative fatality window in colorectal cancer surgery
Source: BJS Open. 2025 Jan 24;9(1):zrae153. doi: 10.1093/bjsopen/zrae153 (PMC11758370; doi:10.1093/bjsopen/zrae153)
Supplement: zrae153_Supplementary_Data [file zrae153_supplementary_data.zip › Annex.zip!/ReadBeforeUse.pdf]

The MATLAB files provided here address some of the primary problem discussed in the article. They are not designed to solve a general problem, and modifying the input or structure may lead to unexpected errors. Users may use and modify the files at their own risk.

Included with the files is 40k of simulated data that mimics real data behavior, illustrating model lack of fit.

The script named “**ScriptOne**” does not use any patients’ variables and estimates the parameters $A = e^{a_0}, B = e^{b_0}$, and $\tau = e^{\tau_0}$ with confidence intervals (CI). It also plots the model hazard versus the smoothed hazard, and the model cumulative hazard versus the empirical cumulative hazard.

The script named “**ScriptTwo**” calculates the P-values for the overall test and the parameters a_1, b_1 and τ_1 for an imaginary patient characteristic coded with a dummy variable in the simulated data. The estimated model is evaluated with plots.

SimDataLOF40k.mat contains the simulated data:

- **Column 1**: 1 = Dead, 0 = Censored
- **Column 2**: Times for events (capped at 180 in the data)
- **Column 3**: Factor indicator (0/1)

Functions:

- **Hazard.m**: The hazard function as described in the appendix.
- **CumHazard.m**: The cumulative hazard function as described in the appendix.
- **Smooth.m**: Used to smooth a NA-estimation of the cumulative hazards.

The nine functions of type “MinusLogMLE_XXXXXX” are used by the optimizer to construct the required optimization problems. The main structure in the files can be extended to cover more covariates with some cut-and-paste programming.

For publication purposes, the .m files have been converted to PDF, and the .mat file to an Excel file.
