# Supplementary material for: Estimation of the postoperative fatality window in colorectal cancer surgery
Source: BJS Open. 2025 Jan 24;9(1):zrae153. doi: 10.1093/bjsopen/zrae153 (PMC11758370; doi:10.1093/bjsopen/zrae153)
Supplement: zrae153_Supplementary_Data [file zrae153_supplementary_data.zip › Annex.zip!/ScriptOne.pdf]

```
clear all
close all

% This script
% estimates the parameters A=e^a0 B=e^b0 and T=e^t0 with CI
% plot the model hazard vs the smoothed hazard
% plot the model cumulative hazard vs the empirical cumulative hazard

%User settings
ts=3       %Time when model starts
Tmax=180    %Time endpoint

%Loading the data
%If data is changed user may need to change optimization settings and
%plotting ranges

load SimDataLOF40k.mat
Data=SimDataLOF40k;



%Data Column1: 1=Dead 0=Censored
%Data Column2: Times for events (Cut at 180 in Data)
%Data Column3: Factor indicator 0-1. (Not used here)

% Ploting the smoothed empirical hazard
figure(1)
% Only use data bigger than ts
alus=(Data(:,2)>=ts);

[xS,yS]=Smooth(Data(find(alus),2),Data(find(alus),1)==0,3,Tmax,1);
leg1 = plot(xS,yS,'r--')
hold on

%Start values in optimization
a0=8e-4;
b0=2e-4;
tau0=30;
par=[a0,b0,tau0];

%Lower and upper bounds in optimization
lb=[0;0;0];
ub=[20e-4;10e-4;100];

options=optimset('MaxFunEvals',10000,'MaxIter',10000); %change if iteration
gets stuck

FunABT = @(x) MinusLogMLE_ABT(x,Data,ts,Tmax);

prob1=createOptimProblem('fmincon','x0',par,'objective',FunABT,'lb',lb,'ub',u
b,'options',options)
```

```
ms = MultiStart('Display','iter')

[Paropt,pLHopt]=run(ms,prob1,20)

%Below for plotting the estimated hazard
aG1=Paropt(1);
bG1=Paropt(2);
tG1=Paropt(3);

x1=[ts,tG1+ts,Tmax];
y1=[aG1,bG1,bG1];
leg2=plot(x1,y1,'r')
axis([0,Tmax,0,aG1])
title('Hazard (No Covariate)')
xlabel('Time')
ylabel(['Hazard'])
legend([leg1 leg2],'Empirical hazard','Model hazard')


%Below for ploting CumHaz and Aalen estimator
%User may want to zoom in picture to locate missfit.
%Look at area near zero to detect missfit if ts=0.

figure(2)

% Only use data bigger than ts
alus=(Data(:,2)>=ts);

[f,time,flo,fup]=ecdf(Data(find(alus),2),'function','cumulative
hazard','censoring',Data(find(alus),1)==0,'bounds','on');
leg3=stairs(time,f,'r');
hold on;
leg4=stairs(time,flo,'r--'); stairs(time,fup,'r--');

x=0:0.01:Tmax;
y=CumHazard(x,Paropt(1),Paropt(2),Paropt(3),ts,Tmax);
leg5=plot(x,y,'k')
legend([leg3 leg4 leg5],'Empirical cumulative hazard','Empirical 95%CI
cumulative hazard','Model cumulative hazard')
ylabel('Cumulative hazard')
xlabel('Time Days')
title('Model evaluation with cumulative hazard')


%Below CI for A B and Tau

%Increace number of MS-iterations if algoritm gets stuck in suboptimal
solution.
%May need to change iteration area if changing Ts and Tmax or Data

%----------------------------------------------------------
% CI for Tau
```

```
x0=[Paropt(1),Paropt(2)]; %start values for A and B
lb=[0;0]; %lower bound in opt
ub=[20e-4;10e-4]; %upper bound in opt

% Vector T around opt-value for PLH-CI-calculations
MinT0=max(Paropt(3)-5,0);
MaxT0=min(Paropt(3)+10,100);
T0=[MinT0:0.5:MaxT0];


for i=1:length(T0)

    FunABX = @(x) MinusLogMLE_ABX(x,T0(i),Data,ts,Tmax);


probT0=createOptimProblem('fmincon','x0',x0,'objective',FunABX,'lb',lb,'ub',u
b,'options',options)

   [ParoptT,pLHoptT]=run(ms,probT0,5);

   ToptAB(i,:)=ParoptT;
   ToptPLH(i)= pLHoptT;
end


%-----------------------------------------------------------------------------
%  CI for B


x0=[Paropt(1),Paropt(3)]; %start values for A and T)
lb=[0;0];
ub=[20e-4;100];

MinB0=Paropt(2)*0.9 %change limit if needed
MaxB0=Paropt(2)*1.1; %change limit if needed
B0=linspace(MinB0,MaxB0,20)

for i=1:length(B0)

    FunAXT =  @(x) MinusLogMLE_AXT(x,B0(i),Data,ts,Tmax);


probB0=createOptimProblem('fmincon','x0',x0,'objective',FunAXT,'lb',lb,'ub',u
b,'options',options)

   [ParoptB,pLHoptB]=run(ms,probB0,5);

   BoptAT(i,:)=ParoptB;
   BoptPLH(i)= pLHoptB ;
end



%-----------------------------------------------------------------------------
```

```
%  CI for A

x0=[Paropt(2),Paropt(3)]; %start values for B and T
lb=[0;0];
ub=[10e-4;100];

MinA0=Paropt(1)*0.7;
MaxA0=Paropt(1)*1.2;
A0=linspace(MinA0,MaxA0,20)

for i=1:length(A0)

    FunXBT =  @(x) MinusLogMLE_XBT(x,A0(i),Data,ts,Tmax);


probA0=createOptimProblem('fmincon','x0',x0,'objective',FunXBT,'lb',lb,'ub',u
b,'options',options)

   [ParoptA,pLHoptA]=run(ms,probA0,5);

   AoptBT(i,:)=ParoptA;
   AoptPLH(i)= pLHoptA ;
end




%----------------------------------------------------------------
%Press the intersection of lines to the left than to the right
%----------------------------------------------------------------
% CI for B plot
figure
plot(B0,-BoptPLH)
title('Par:B Press in Plot for lower and then upper limit')
ylabel('Log(PLH) and CI limit line')
xlabel('Parameter value')
hold on
plot(Paropt(2),-pLHopt,'r*')
Ygrans=-pLHopt-chi2inv(0.95,1)/2;
plot([MinB0,MaxB0],[Ygrans,Ygrans])

[BlowUp,tmp]=ginput(2);


CI_B=BlowUp;
PE_B=Paropt(2);


%----------------------------------------------------
% CI for T plot


figure
plot(T0,-ToptPLH)
```

```
title('Par:T Press in Plot for lower and then upper limit')
ylabel('Log(PLH) and CI limit line')
xlabel('Parameter value')

hold on
plot(Paropt(3),-pLHopt,'r*')
Ygrans=-pLHopt-chi2inv(0.95,1)/2;
plot([MinT0,MaxT0],[Ygrans,Ygrans])

[TlowUp,tmp]=ginput(2);
CI_T=TlowUp;
PE_T=Paropt(3);

%----------------------------------------------------
% CI for A plot

figure
plot(A0,-AoptPLH)
title('Par:A Press in Plot for lower and then upper limit')
ylabel('Log(PLH) and CI limit line')
xlabel('Parameter value')
hold on

plot(Paropt(1),-pLHopt,'r*')
Ygrans=-pLHopt-chi2inv(0.95,1)/2;
plot([MinA0,MaxA0],[Ygrans,Ygrans])

[AlowUp,tmp]=ginput(2);

CI_A=AlowUp;
PE_A=Paropt(1);


%Typ={'Point Estimate','CI95%-low','CI95%-high'}';

PAR={'A','B','Tau','Tfas=Ts+Tau'}';
Estimate=[PE_A,PE_B,PE_T,PE_T+ts]';
CI_low=[CI_A(1),CI_B(1),CI_T(1),CI_T(1)+ts]';
CI_high=[CI_A(2),CI_B(2),CI_T(2),CI_T(2)+ts]';

T=table(PAR,Estimate,CI_low,CI_high)
```

Published with MATLAB® R2024b
