# Supplementary material for: Estimation of the postoperative fatality window in colorectal cancer surgery
Source: BJS Open. 2025 Jan 24;9(1):zrae153. doi: 10.1093/bjsopen/zrae153 (PMC11758370; doi:10.1093/bjsopen/zrae153)
Supplement: zrae153_Supplementary_Data [file zrae153_supplementary_data.zip › Annex.zip!/ScriptTwo.pdf]

```
clear all
close all

% This script calculates the Pvalues for
% the overall test
% the a1 parameter
% the b1 parameter
% the t1 parameter
% and makes plots comparing model and empirical hazards

load SimDataLOF40k.mat
Data=SimDataLOF40k;

%Data Column1: 1=Dead 0=Censored
%Data Column2: Times for event (Cut at 180 in Data)
%Data Column3: Factor indicator 0/1

%user settings

ts=3
Tmax=180
NumMS=20; %Number or multistart runs.

%Some start point for iteration
a0=-7;
a1=0;
b0=-8;
b1=0;
t0=3;
t1=0;

parStart=[a0,a1,b0,b1,t0,t1]; %Opt start values

%Limit for optimization
%Change if change Data
%Make wider if CI search
a0min=-12;
a0max=-2;
a1min=-1;
a1max=1;

b0min=-12;
b0max=-5;
b1min=-1;
b1max=1;

t0min=1;
t0max=5;
t1min=-1;
t1max=1;
```

```
%--------- Estimates no restrictions HA (nr1) --------------------

lb=[a0min;a1min;b0min;b1min;t0min;t1min];
ub=[a0max;a1max;b0max;b1max;t0max;t1max];

parHA=parStart;
options=optimset('MaxFunEvals',10000,'MaxIter',10000); %change if iteration
gets stuck

FunAABBTT = @(x) MinusLogMLE_AABBTT(x,Data,ts,Tmax);

prob1=createOptimProblem('fmincon','x0',parHA,'objective',FunAABBTT,'lb',lb,'
ub',ub,'options',options)

ms = MultiStart('Display','iter')
[ParoptHAms,pLHoptHAms]=run(ms,prob1,NumMS)

%-----------------------------------------------



%---------- Picture of estimates ---------------

figure
aG1=exp(ParoptHAms(1));
aG2=exp(ParoptHAms(1)+ParoptHAms(2));
bG1=exp(ParoptHAms(3));
bG2=exp(ParoptHAms(3)+ParoptHAms(4));
tG1=ts+exp(ParoptHAms(5));
tG2=ts+exp(ParoptHAms(5)+ParoptHAms(6));

x1=[ts,tG1,Tmax];
y1=[aG1,bG1,bG1];
x2=[ts,tG2,Tmax];
y2=[aG2,bG2,bG2];
leg1 = plot(x1,y1,'r')
hold on
leg2 = plot(x2,y2,'b')
axis([0,Tmax,0,max(aG1,aG2)*1.05])
title('Hazards for the baseline and indicated level')
xlabel('Time (Days)')
ylabel(['Hazard'])


DataRef=Data(find( (Data(:,3)==0).*(Data(:,2)>=ts) ),:);
DataInd=Data(find( (Data(:,3)==1).*(Data(:,2)>=ts) ),:);

[xSref,ySref]=Smooth(DataRef(:,2),DataRef(:,1)==0,3,Tmax,1);
[xSind,ySind]=Smooth(DataInd(:,2),DataInd(:,1)==0,3,Tmax,1);

leg3 = plot(xSref,ySref,'r--')
leg4 = plot(xSind,ySind,'b--')

legend([leg1 leg2 leg3 leg4],'Model hazard baseline','Model hazard
```

```
indicated','Empirical hazard baseline','Empirical hazard indicated')


%-----------------------------------------------------
figure

[f,time,flo,fup]=ecdf(DataRef(:,2),'function','cumulative
hazard','censoring',DataRef(:,1)==0,'bounds','on');
leg5=stairs(time,f,'r');
hold on;
leg6=stairs(time,flo,'r--'); stairs(time,fup,'r--');

x=0:0.01:Tmax;
y=CumHazard(x,exp(ParoptHAms(1)),exp(ParoptHAms(3)),exp(ParoptHAms(5)),ts,Tma
x);
leg7=plot(x,y,'k')
legend([leg5 leg6 leg7],'Empirical cumulative hazard','Empirical 95%CI
cumulative hazard','Model cumulative hazard')
ylabel('Cumulative hazard')
xlabel('Time Days')
title('Cumulative hazard plot at baseline level')

figure

[f,time,flo,fup]=ecdf(DataInd(:,2),'function','cumulative
hazard','censoring',DataInd(:,1)==0,'bounds','on');
leg8=stairs(time,f,'r');
hold on;
leg9=stairs(time,flo,'r--'); stairs(time,fup,'r--');

x=0:0.01:Tmax;
y=CumHazard(x,exp(ParoptHAms(1)+ParoptHAms(2)),exp(ParoptHAms(3)+ParoptHAms(4
)),exp(ParoptHAms(5)+ParoptHAms(6)),ts,Tmax);
leg10=plot(x,y,'k')
legend([leg8 leg9 leg10],'Empirical cumulative hazard','Empirical 95%CI
cumulative hazard','Model cumulative hazard')
ylabel('Cumulative hazard')
xlabel('Time Days')
title('Cumulative hazard plot at indicated level')


%-- overall test (nr2) ---------------------------

lb3par=[a0min;b0min;t0min];
ub3par=[a0max;b0max;t0max];

FunA0B0T0 = @(x) MinusLogMLE_A0B0T0(x,Data,ts,Tmax);

parH0=[a0,b0,t0];

prob2=createOptimProblem('fmincon','x0',parH0,'objective',FunA0B0T0,'lb',lb3p
ar,'ub',ub3par,'options',options)

[ParoptH0ms2,pLHoptH0ms2]=run(ms,prob2,NumMS);
```

```
kvantil=-(pLHoptHAms-pLHoptH0ms2)*2;
P_overall=chi2cdf(kvantil,3,'upper')

%--------------------------------------------------



%---H0: a1=0 (nr3) --------------------------------

parH0=[a0,b0,b1,t0,t1]; %a1 set to 0

lba1=[a0min;b0min;b1min;t0min;t1min];
uba1=[a0max;b0max;b1max;t0max;t1max];

FunA0BBTT = @(x) MinusLogMLE_A0BBTT(x,Data,ts,Tmax);

prob3=createOptimProblem('fmincon','x0',parH0,'objective',FunA0BBTT,'lb',lba1
,'ub',uba1,'options',options)

[ParoptH0ms3,pLHoptH0ms3]=run(ms,prob3,NumMS);
kvantil=-(pLHoptHAms-pLHoptH0ms3)*2;
P_a1=chi2cdf(kvantil,1,'upper')

%--------------------------------------------------




%---H0: b1=0 (nr4)---------------------------------

parH0=[a0,a1,b0,t0,t1]; %b1 set to 0

lbb1=[a0min;a1min;b0min;t0min;t1min];
ubb1=[a0max;a1max;b0max;t0max;t1max];

FunAAB0TT = @(x) MinusLogMLE_AAB0TT(x,Data,ts,Tmax);


prob4=createOptimProblem('fmincon','x0',parH0,'objective',FunAAB0TT,'lb',lbb1
,'ub',ubb1,'options',options)

[ParoptH0ms4,pLHoptH0ms4]=run(ms,prob4,NumMS);
kvantil=-(pLHoptHAms-pLHoptH0ms4)*2;
P_b1=chi2cdf(kvantil,1,'upper')

%--------------------------------------------------



%---H0: t1=0 (nr5) -------------------------------

parH0=[a0,a1,b0,b1,t0]; %t1 set to 0

lbt1=[a0min;a1min;b0min;b1min;t0min];
```

```
ubt1=[a0max;a1max;b0max;b1max;t0max];


FunAABBT0 = @(x) MinusLogMLE_AABBT0(x,Data,ts,Tmax);


prob5=createOptimProblem('fmincon','x0',parH0,'objective',FunAABBT0,'lb',lbt1
,'ub',ubt1,'options',options)

[ParoptH0ms5,pLHoptH0ms5]=run(ms,prob5,NumMS)
kvantil=-(pLHoptHAms-pLHoptH0ms5)*2;
P_t1=chi2cdf(kvantil,1,'upper')

%--------------------------------------------------



Hypothesis={'Overall','H0: a1=0','H0: b1=0','H0: t1=0'}';
Pvalue=[P_overall,P_a1,P_b1,P_t1]';
T=table(Hypothesis,Pvalue)
```

Published with MATLAB® R2024b
