# Supplementary material for: Estimation of the postoperative fatality window in colorectal cancer surgery
Source: BJS Open. 2025 Jan 24;9(1):zrae153. doi: 10.1093/bjsopen/zrae153 (PMC11758370; doi:10.1093/bjsopen/zrae153)
Supplement: zrae153_Supplementary_Data [file zrae153_supplementary_data.zip › Annex.zip!/Smooth.pdf]

```
function [x,y]=Smooth(time,censor,sigma,Tmax,edgecorr)
% [x,y]=Smooth(tider,censor,sigma,Tmax,edgecorr)
% Smooths a Hazard. Uses Normal shaped kernal that operates on the
% increments of the Aalen cumulative hazard
% x and y are outout vectors
% time: Vector of times
% censor: Indicator what times are censored
% sigma: Amount of smooting
% Tmax: Max value for x
% edgecorr: User decide number of sigma removed in output due to eage bias.

[y,x]=ecdf(time,'function','cumulative hazard','censoring',censor);

yspikar=[y;y(length(y))]-[0;y];
xspikar=[x;x(length(x))];

xind=[0:Tmax];

for i=1:length(xspikar)
    SmoothMatris(i,:)=normpdf(xind,xspikar(i),sigma)*yspikar(i);
end

Smooth=sum(SmoothMatris);

%Below remove the times near the eges

index= find((xind>min(xspikar)+edgecorr*sigma) .* (xind<max(xspikar)-
edgecorr*sigma));
x=xind(index);
y=Smooth(index);


end
```

Published with MATLAB® R2024b
