# Supplementary material for: Estimation of the postoperative fatality window in colorectal cancer surgery
Source: BJS Open. 2025 Jan 24;9(1):zrae153. doi: 10.1093/bjsopen/zrae153 (PMC11758370; doi:10.1093/bjsopen/zrae153)
Supplement: zrae153_Supplementary_Data [file zrae153_supplementary_data.zip › Annex.zip!/CumHazard.pdf]

```
function [ cumhaz ] = CumHazard(t,a,b,tau,ts,tmax)
% [ cumhaz ] = CumHazard(t,a,b,tau,ts,tmax)
% Returns the cumulative hazard
% time: t  (Can be vector)
% Parameters: a b tau (scalars or vectors of length(t))
% t=vector
% a b c tau scalär
% ts: Tstart set by user
% tmax: Tmax set by user
% Returns 0 if t<ts
% Returns CumHazard(tmax) if t>tmax

tfas=ts+tau;
%time indicators
I1=t<ts;
I2=[t>=ts].*[t<tfas];
I3=[t>=tfas].*[t<=tmax];
I4=t>tmax;


part1=(a.*(t-ts)-((a-b)./(tfas-ts)).*(t-ts).^2/2).*I2;
part2=( (tfas-ts).*(a+b)/2+b.*(t-tfas)).*I3;
part3=( (tfas-ts).*(a+b)/2+b.*(tmax-tfas)).*I4;

cumhaz=part1+part2+part3;
end
```

Published with MATLAB® R2024b
