# Supplementary material for: Estimation of the postoperative fatality window in colorectal cancer surgery
Source: BJS Open. 2025 Jan 24;9(1):zrae153. doi: 10.1093/bjsopen/zrae153 (PMC11758370; doi:10.1093/bjsopen/zrae153)
Supplement: zrae153_Supplementary_Data [file zrae153_supplementary_data.zip › Annex.zip!/Hazard.pdf]

```
function [ hazard ] = Hazard(t,a,b,tau,ts,tmax)
% [ hazard ] = Hazard(t,a,b,tau,ts,tmax)
% Returns the value of the hazard
% Time: t (Can be vector)
% Parameters: a b tau (scalars or vectors of length(t))
% ts: Tstart set by user
% tmax: Tmax set by user
% Returns NaN if t<ts or t>tmax


tfas=ts+tau;
%time indicators
I1=t<ts;
I2=[t>=ts].*[t<tfas];
I3=[t>=tfas].*[t<=tmax];
I4=t>tmax;

%Values of hazard
part1=(a-((a-b)./(tfas-ts).*(t-ts))).*I2;
part2=b.*I3;
part2(find(I1|I4))=NaN;

hazard=part1+part2;


end
```

Published with MATLAB® R2024b
