# Supplementary material for: Estimation of the postoperative fatality window in colorectal cancer surgery
Source: BJS Open. 2025 Jan 24;9(1):zrae153. doi: 10.1093/bjsopen/zrae153 (PMC11758370; doi:10.1093/bjsopen/zrae153)
Supplement: zrae153_Supplementary_Data [file zrae153_supplementary_data.zip › Annex.zip!/MinusLogMLE_AABBT0.pdf]

```
function [ out ] = MinusLogMLE_AABBT0(par,Data,ts,Tmax)
% [ out ] = MinusLogMLE_AABBT0(par,Data,ts,Tmax)
% Needed to solve the hypothesis
% H0: t1=0
% This function is used as middle step for the function handeling used to
% create a optimization problem "createOptimProblem"
% Data(:,1)= event (1=dead 0=censored)
% data(:,2)= time
% data(:,3)= kovariat
% par(1)=a0
% par(2)=a1
% par(3)=b0
% par(4)=b1
% par(5)=t0
% ts: Tstart set by user
% Tmax: Tmax set by user
% Parameters t1=0


parAABBTT=[par(1),par(2),par(3),par(4),par(5),0];

out=MinusLogMLE_AABBTT(parAABBTT,Data,ts,Tmax);

end
```

Published with MATLAB® R2024b
