# Supplementary material for: Estimation of the postoperative fatality window in colorectal cancer surgery
Source: BJS Open. 2025 Jan 24;9(1):zrae153. doi: 10.1093/bjsopen/zrae153 (PMC11758370; doi:10.1093/bjsopen/zrae153)
Supplement: zrae153_Supplementary_Data [file zrae153_supplementary_data.zip › Annex.zip!/MinusLogMLE_AABBTT_3.pdf]

```
function [ out ] = MinusLogMLE_AABBTT(par,Data,ts,Tmax)
% [ out ] = MinusLogMLE_AABBTT(par,Data,ts,Tmax)
% Returns the minus log(ML)-function
% This function is used as middle step for the function handeling used to
% create a optimization problem "createOptimProblem"
% Data(:,1)= event (1=dead 0=censored)
% data(:,2)= time
% data(:,3)= kovariat
% par(1)=a0
% par(2)=a1
% par(3)=b0
% par(4)=b1
% par(5)=t0
% par(6)=t1
% ts: Tstart set by user
% Tmax: Tmax set by user


%Cuts Data at Tmax
censurobs=find(Data(:,2)>=Tmax);
Data(censurobs,1)=0;    %Death later than Tmax is censored
Data(censurobs,2)=Tmax; %Time later than Tmax is Tmax

% bigger than ts
alus=(Data(:,2)>=ts);

% Dead
dead=(Data(:,1)==1);


% Times bigger than ts and dead
td=Data(find(dead.*alus),2);
DeadAlusInd=find(dead.*alus);

%times bigger than ts
tu=Data(find(alus),2);
AlusInd=find(alus);

%Vektors a b t for used observations
a=exp( par(1)+par(2).*Data(AlusInd,3) );
b=exp( par(3)+par(4).*Data(AlusInd,3) );
Tau=exp( par(5)+par(6).*Data(AlusInd,3) );


%Vektorer a b t for used and dead observations
ad=exp( par(1)+par(2).*Data(DeadAlusInd,3) );
bd=exp( par(3)+par(4).*Data(DeadAlusInd,3) );
Taud=exp( par(5)+par(6).*Data(DeadAlusInd,3) );

del1=sum(log(Hazard(td,ad,bd,Taud,ts,Tmax)));
del2=sum(CumHazard(tu,a,b,Tau,ts,Tmax));
logMLE=del1-del2;
```

```
out=-logMLE;

end
```

Published with MATLAB® R2024b
