# Supplementary material for: Estimation of the postoperative fatality window in colorectal cancer surgery
Source: BJS Open. 2025 Jan 24;9(1):zrae153. doi: 10.1093/bjsopen/zrae153 (PMC11758370; doi:10.1093/bjsopen/zrae153)
Supplement: zrae153_Supplementary_Data [file zrae153_supplementary_data.zip › Annex.zip!/MinusLogMLE_ABT.pdf]

```
function [ out ] = MinusLogMLE_ABT(par,Data,ts,Tmax)
% [ out ] = MinuslogMLE_ABT(par,Data,ts,Tmax)
% Returns the minus log(ML)-function
% This function is used as middle step for the function handeling used to
% create a optimization problem "createOptimProblem"
% Data(:,1)= event (1=dead 0=censored)
% data(:,2)= time
% par(1):A=e^a0
% par(2):B=e^b0
% par(3):T=e^t0
% ts: Tstart set by user
% Tmax: Tmax set by user

%Cuts Data at Tmax
censurobs=find(Data(:,2)>=Tmax);
Data(censurobs,1)=0;    %Death later than Tmax is censored
Data(censurobs,2)=Tmax; %Time later than Tmax is Tmax

% bigger than ts
alus=(Data(:,2)>=ts);
% Dead
dead=(Data(:,1)==1);

% Times bigger than ts and dead
td=Data(find(dead.*alus),2);

%times bigger than ts
tu=Data(find(alus),2);

a=par(1);
b=par(2);
Tfas=par(3);

del1=sum(log(Hazard(td,a,b,Tfas,ts,Tmax)));
del2=sum(CumHazard(tu,a,b,Tfas,ts,Tmax));
logMLE=del1-del2;

out=-logMLE;
end
```

Published with MATLAB® R2024b
