# Supplementary material for: Estimation of the postoperative fatality window in colorectal cancer surgery
Source: BJS Open. 2025 Jan 24;9(1):zrae153. doi: 10.1093/bjsopen/zrae153 (PMC11758370; doi:10.1093/bjsopen/zrae153)
Supplement: zrae153_Supplementary_Data [file zrae153_supplementary_data.zip › Annex.zip!/MinusLogMLE_ABX.pdf]

```
function [ out ] = MinusLog_ABX(parAB,parT,Data,Ts,Tmax)
% [ out ] = MinusLog_ABX(parAB,parT,Data,Ts,Tmax)
% Returns the minus log(ML)-function for fix Tau
% This function is used as middle step for the function handeling used to
% create a optimization problem "createOptimProblem"

parABT=[parAB(1),parAB(2),parT];

out=MinusLogMLE_ABT(parABT,Data,Ts,Tmax);

end
```

Published with MATLAB® R2024b
